# The “Are You OK?” Paradox: A Scoping Review of Nocebo and Negative Suggestion in Healthcare Communication

**DOI:** 10.3390/dj14050274

**Published:** 2026-05-06

**Authors:** Orion K. O’Brien, Christopher C. Donnell

**Affiliations:** 1 Department of Paediatric Dentistry, Charles Clifford Dental Hospital, Sheffield Teaching Hospitals NHS Foundation Trust, Sheffield S10 2SZ, UK; 2 Academic Unit of Oral Health, Dentistry and Society, School of Clinical Dentistry, University of Sheffield, Sheffield S10 2TA, UK

**Keywords:** nocebo, expectancy, clinical communication, behaviour support, reassurance, paediatric dentistry, sedation

## Abstract

**Background:** Nocebo effects are described as adverse symptoms arising from negative expectations rather than direct physiological harm, and are increasingly recognised across healthcare. While traditionally examined within pharmacological trials, emerging literature suggests that nocebo effects are shaped by broader interactional, situational, and communicative processes. In dentistry and paediatric care, where behaviour support and reassurance are central to practice, these mechanisms remain under-synthesised. **Objectives:** This scoping review aimed to map how nocebo effects are conceptualised across healthcare literature, with particular attention to the role of communication, reassurance, and behaviour support, and to explore how these mechanisms are discussed in paediatric, procedural, and dental contexts. **Methods:** An interpretive scoping review was conducted in line with JBI guidance and PRISMA-ScR reporting standards. Multidisciplinary literature spanning experimental, clinical, ethical, and applied domains was systematically identified and charted. Studies were grouped using a conceptual framework encompassing expectancy, learning, communication-mediated, ethical, and contextual mechanisms, allowing overlap between categories. **Results:** A large and heterogeneous body of literature was identified, with most studies conceptualising nocebo effects through overlapping mechanisms rather than discrete pathways. Expectancy and learning processes formed a foundational substrate across contexts, while communication, including framing, tone, reassurance, and checking-in, emerged as an active mechanism shaping symptom perception and vigilance. Ethical discussions highlighted tensions between transparency and potential harm, particularly in consent and risk communication. Paediatric and procedural settings, including dental sedation, were comparatively underrepresented despite features likely to amplify nocebo effects, such as reduced agency and heightened attentional focus. **Conclusions:** Nocebo effects are best understood as interactional phenomena that emerge within everyday clinical encounters. This review highlights the need to critically examine behaviour support practices, including reassurance, that are typically assumed to be benign. Greater conceptual clarity and reflexivity in communication may support future research and training aimed at minimising unintended distress within dental and paediatric care. These findings suggest that routine communication practices, including reassurance and expectation-setting, should be understood as active components of care that can influence patient experience, rather than as neutral or purely supportive interactions.

## 1. Introduction

Nocebo effects are described as “adverse symptoms or experiences arising from negative expectations rather than direct physiological harm”, and are now recognised as a robust and clinically relevant phenomenon across healthcare settings [1,2,3]. Historically, nocebo effects were most clearly documented in pharmacological trials, where adverse events reported in placebo arms drew attention to the influence of expectation, attribution, and contextual framing on symptom experience [4,5,6]. Once considered the “evil twin” of placebo, nocebo responses are increasingly understood not as rare artefacts of experimental design, but as predictable outcomes of how treatments are framed, communicated, and experienced within clinical encounters [7,8].

Experimental and clinical research consistently demonstrates that expectations, prior learning, and contextual cues can amplify pain, distress, and symptom vigilance even in the absence of pharmacological change [9,10,11]. Critically, nocebo effects are not generated by expectation alone, but are shaped through learning processes, social modelling, and interpersonal interaction—conditioning, observational learning, and verbal suggestion interact dynamically to establish symptom meaning and threat appraisal over time [12,13,14]. These mechanisms are reinforced by emotional memory, anxiety, and attentional focus, which together sustain heightened symptom monitoring and attribution [15,16]. As such, nocebo effects appear to emerge not simply from what clinicians say, but from how, when, and to whom information is delivered.

Communication therefore occupies a central position within nocebo theory. Verbal framing, tone, emphasis, and interpersonal context have all been shown to influence how patients interpret bodily sensations and anticipate outcomes [17,18,19]. Even language intended to promote transparency or reassurance may inadvertently increase vigilance or distress when delivered without sensitivity to prior experience, perceived control, or the immediate encounter context [20,21]. This reframes clinical communication not as a neutral conduit for information, but as an active mechanism within the nocebo process itself [22,23].

Nowhere is this tension more apparent than in routine reassurance. Phrases such as “you’re fine,” or “this won’t hurt,” or repeated check-ins of “are you OK?” are widely assumed to be benign, supportive, and ethically sound. Yet emerging literature suggests that, under certain conditions, repeated reassurance may paradoxically heighten distress by signalling threat, drawing attention to internal sensations, or undermining a patient’s sense of agency [24,25,26]. Rather than soothing, such reassurance may function as a cue that something is or might be wrong, thereby amplifying the very responses it seeks to prevent.

Despite a rapidly expanding evidence base, nocebo research remains fragmented across experimental psychology, medicine, ethics, and applied clinical fields, with limited synthesis of how these mechanisms intersect with behaviour support practices in procedural and paediatric care [16,27,28,29]. While this review does not seek to advance a new theoretical construct, it adopts an interpretive lens to examine how nocebo-related mechanisms are conceptualised and discussed across clinical contexts, with particular attention to how communication and reassurance function within behaviour support practices. The aim was therefore to map and synthesise this literature in order to consider its implications for behaviour support practices, rather than to evaluate intervention effectiveness or synthesise outcomes quantitatively.

## 2. Methods

### 2.1. Review Design

This study was conducted as a scoping review to enable systematic mapping of the breadth, nature, and conceptual framing of literature relating to nocebo effects, negative expectancy, and clinical communication within behaviour support contexts. A scoping approach was considered appropriate given the anticipated heterogeneity of study designs, the inclusion of empirical, theoretical, and ethical literature, and the absence of a single dominant methodological or theoretical framework addressing these phenomena [30,31].

The review was informed by guidance from the Joanna Briggs Institute (JBI) for scoping reviews and is reported in accordance with the PRISMA Extension for Scoping Reviews (PRISMA-ScR) [32].

### 2.2. Review Questions

The review was guided by the following research questions:

1. How are nocebo effects and negative expectancy conceptualised and operationalised within healthcare and behaviour support literature?
2. What forms of verbal or non-verbal communication are associated with the development or amplification of negative expectations or symptom perception?
3. How are these mechanisms discussed in relation to reassurance, behaviour management, or supportive clinical communication?
4. What gaps exist in the application of nocebo theory to paediatric and procedural healthcare contexts?

### 2.3. Information Sources and Search Strategy

A comprehensive search strategy was developed in consultation with an academic health librarian. Electronic searches were conducted across multiple bibliographic databases, including MEDLINE (via Ovid), Embase, PsycINFO, Scopus, and Web of Science.

To ensure a broad capture of relevant conceptual, theoretical, and interdisciplinary literature, searches were not limited to traditional bibliographic databases. Additional searches were undertaken using Google Scholar, Nexis, and EthOS, alongside manual screening of reference lists from included studies. This broader approach was adopted to account for the diverse ways in which nocebo effects, expectation-shaping, and communication-related mechanisms are discussed across clinical, psychological, and behavioural science literature.

Search terms combined controlled vocabulary (e.g., MeSH terms) and free-text keywords relating to nocebo, negative expectancy, verbal suggestion, clinical communication, reassurance, behaviour support, anxiety, and procedural distress. Paediatric-related terms were included to ensure capture of child-focused literature; however, no age limits were applied, and adult and mixed-population studies were retained. The full search strategy for MEDLINE is provided in Table 1, which was adapted as required for each database.

### 2.4. Eligibility Criteria

Eligibility criteria were defined a priori using the Population-Concept-Context (PCC) framework [33]. There was no restriction on study design or date of publication.

#### 2.4.1. Population

Studies involving children, adolescents, or adults were eligible. Literature focusing on healthcare professionals, caregivers, or experimental participants was also included where communication, expectation-setting, or behaviour support formed a central component of the study.

#### 2.4.2. Concept

Studies were eligible if they addressed one or more of the following:

- Nocebo effects or negative expectancy.
- Symptom amplification, anticipatory anxiety, or expectation-driven responses.
- Verbal or non-verbal clinical communication, including reassurance, framing, or warning language.
- Behaviour support or behaviour management strategies within healthcare settings.

Studies focusing solely on pharmacological adverse effects without reference to communication or expectancy were excluded.

#### 2.4.3. Context

Healthcare and clinical contexts were included, encompassing medical, dental, psychological, nursing, and allied health settings. Experimental or laboratory-based studies were included where findings were relevant to clinical communication or expectancy formation. Studies conducted exclusively in non-healthcare or marketing contexts were excluded.

#### 2.4.4. Additional Criteria

- Only articles published in English were included (and where no translation into English was available, articles were excluded).
- Grey literature was accepted, as defined by GreyNet International [34].
- Conference abstracts without sufficient methodological detail were excluded.
- Where no access past the title and abstract was possible, e.g., due to a paid firewall, these articles were excluded.

### 2.5. Study Selection and Data Extraction

All retrieved records were imported into Microsoft Excel (Microsoft Excel for Mac Version 16.101.3 [25100321]), and duplicates were removed prior to screening. Titles and abstracts were screened against the eligibility criteria, followed by full-text review of potentially relevant articles. Screening was conducted iteratively by OKO and CCD, allowing refinement of eligibility criteria as familiarity with the literature increased. Where uncertainty arose regarding inclusion, decisions were resolved through discussion between the authors.

Data were extracted using a purpose-designed data extraction spreadsheet, piloted on a subset of included studies and refined iteratively [35]. The final data extraction headings are shown in Table 2.

### 2.6. Data Synthesis

Given the expected heterogeneity of study designs, populations, and outcomes, a narrative and thematic synthesis approach was adopted [36]. Extracted data were grouped according to conceptual focus and clinical context, allowing patterns in how nocebo-related mechanisms were described, operationalised, and discussed to be explored.

No formal quality appraisal was undertaken, consistent with scoping review methodology [37]. However, methodological limitations were noted where relevant to interpretation, and gaps in the existing literature were identified.

## 3. Results

The database searches identified 2625 records, with an additional 1435 records identified through other sources, including Google Scholar, Nexis, EthOS, and citation searching. Following removal of duplicates and records excluded prior to screening, 768 records were screened at title and abstract level. Of these, 614 records were excluded primarily due to lack of relevance to the review concept or clinical context.

**Figure 1:**
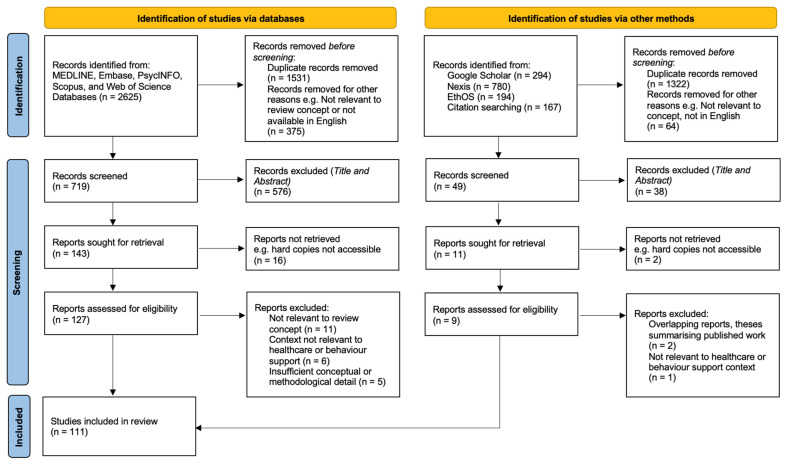
PRISMA-ScR flow diagram of study selection.

A total of 154 reports were sought for full-text retrieval, of which 18 could not be accessed, leaving 136 reports assessed for eligibility. Following full-text review, 25 reports were excluded for reasons including lack of relevance to nocebo-related mechanisms, contexts outside healthcare or behaviour support, or insufficient conceptual or methodological detail. In total, 111 studies met the inclusion criteria and were included in the final synthesis. The study selection process is outlined using a PRISMA-ScR flow diagram—Figure 1.

### 3.1. Characteristics of Included Studies

The included literature comprised a heterogeneous body of work spanning experimental, clinical, review, and ethics-focused publications. Study designs were predominantly experimental or observational, commonly employing laboratory-based pain paradigms, conditioning protocols, or expectancy manipulations to examine nocebo-related outcomes. In addition, narrative reviews, systematic reviews, meta-analyses, and conceptual or ethical commentaries were well represented, reflecting sustained interest in nocebo phenomena across methodological traditions [5,29,38].

Adult populations predominated across the dataset. Experimental studies frequently recruited healthy adult volunteers, while clinical studies were most often situated within pain medicine, psychiatry, anaesthesia, and other procedural healthcare contexts. Paediatric-focused studies were comparatively sparse and were typically observational, qualitative, or applied in nature, with an emphasis on communication practices, preparatory information, or procedural experiences rather than experimentally induced nocebo responses [39,40,41].

The literature spanned a wide range of clinical and applied domains. Pain and analgesia were the most frequently examined outcomes, including musculoskeletal pain, experimental pain models, post-procedural pain, and chronic pain conditions. Other contexts included dentistry, surgery, anaesthesia, psychiatry, rehabilitation, and healthcare decision-making, particularly in relation to informed consent and risk communication [42,43,44]. A smaller subset of studies examined nocebo-related mechanisms in non-clinical or quasi-clinical settings, such as educational, simulated, or experimental communication environments.

Terminology and conceptual framing varied across disciplines. While many studies explicitly used the term nocebo, others examined closely related constructs, including negative expectancy effects, adverse suggestion, symptom amplification, or communication-induced harm, without consistently applying nocebo terminology. This variation appeared to reflect disciplinary differences in language and theoretical emphasis rather than substantive disagreement regarding underlying mechanisms [4,17,45]. A summary of key study characteristics is provided in Table 3.

### 3.2. Conceptualisations of Nocebo Effects Across Healthcare Contexts

#### 3.2.1. Nocebo as Expectancy-Driven Symptom Amplification

Across the included literature, nocebo effects were most commonly conceptualised as expectancy-driven amplification of symptoms, whereby negative expectations (formed through verbal suggestion), information framing, or prior experience, alter symptom perception and reporting in the absence of direct physiological harm. This framing was evident across experimental, applied, and review-based studies and represented the most prevalent conceptual framing across the dataset [5,45,61,71,101,134,135].

Studies consistently described nocebo responses as arising from anticipatory cognitive processes, in which expectations regarding pain, discomfort, performance, or adverse effects influenced subsequent symptom experience. Experimental and translational work demonstrated that negative expectations increased symptom intensity, persistence, or task impairment even when underlying stimuli, treatments, or procedures were unchanged, positioning expectancy as a unifying mechanism across clinical contexts [5,45,61,71,101,135].

#### 3.2.2. Nocebo as Learning, Conditioning, and Prior Experience

A closely related conceptualisation framed nocebo effects as arising through learning, conditioning, and prior experience, whereby previous symptom encounters, repeated exposure to negative cues, or observation of others’ responses shape subsequent symptom perception and reporting. Across the literature, nocebo responses were described not only as momentary expectancy effects, but as learned psychobiological responses reinforced over time through associative learning, memory, and experiential history [9,13,45,61,81,87,88,91].

Experimental, mechanistic, and clinical studies demonstrated that learning histories modulated later symptom reporting even when subsequent stimuli or interventions were unchanged. Within applied healthcare contexts, accumulated treatment experiences and repeated negative messaging were described as conditioning future expectations, symptom vigilance, and distress, including within dentistry- and procedure-relevant settings [7,13,15,18,22,43,44,45,60,77,81,85,87,88,91,110,136,137].

#### 3.2.3. Nocebo as Adverse-Event Misattribution and Risk Communication

Another conceptualisation framed nocebo effects as arising through adverse-event misattribution and risk communication, whereby benign, unrelated, or expected symptoms are attributed to treatment as a consequence of how risks and side effects are communicated. Within this framing, nocebo effects were understood as interpretive phenomena shaped by how bodily sensations are appraised in light of clinician communication or written information [5,15,45,137,138].

Across experimental and clinical studies, negatively framed risk information, detailed side-effect lists, and emphasis on potential harm were associated with increased symptom reporting, particularly for non-specific or ambiguous symptoms, even when pharmacological or procedural exposure was unchanged. In applied healthcare contexts, repeated emphasis on risk or frequent checking-in regarding side effects was described as reinforcing symptom salience and uncertainty, including in medication- and procedure-adjacent care and within dentistry-relevant literature [5,15,18,44,45,87,88,137,138].

#### 3.2.4. Nocebo as a Communication-Mediated and Relational Phenomenon

A further conceptualisation framed nocebo effects as communication-mediated phenomena emerging through clinical interaction, rather than information content alone. Within this framing, nocebo responses were shaped by clinician language, tone, repetition, non-verbal cues, and the broader interactional setting in which information was delivered [17,18,44,45,109,137].

Across experimental, clinical, and applied studies, nocebo effects were described as arising through interactional processes that influenced patients’ interpretation of bodily sensations, emotional responses, and sense of safety. Communication styles emphasising monitoring, risk, or uncertainty were associated with heightened symptom salience and distress, particularly in contexts characterised by reduced patient agency, heightened sensory focus, or procedural constraint [3,4,8,15,18,41,44,45,59,77,87,88,112,113,137,138].

#### 3.2.5. Conceptual Heterogeneity and Inconsistent Terminology

Across the included literature, substantial conceptual heterogeneity was evident in how nocebo effects were defined, operationalised, and discussed. Closely related mechanisms, including expectancy, learning, misattribution, and communication, were frequently labelled differently or positioned at different explanatory levels, resulting in blurred conceptual boundaries across disciplines and study designs [2,18,45,137].

Terminological inconsistency was particularly apparent in the interchangeable use of terms such as nocebo effects, negative expectancy effects, contextual adverse effects, and communication-related symptom amplification. Several reviews cautioned that rigid separation of these constructs may obscure their dynamic interplay within real-world clinical encounters, where expectation, learning, communication, and context frequently co-occur and reinforce one another [18,45,109,137,138].

### 3.3. Communication, Reassurance, and Expectation-Setting Mechanisms

#### 3.3.1. Pain, Symptom Monitoring, and Chronic Conditions

Across the included literature, nocebo effects were most frequently examined in contexts involving pain, ongoing symptom monitoring, and chronic or recurrent conditions. In these settings, nocebo-related processes were commonly identified through heightened symptom reporting, increased pain intensity, or amplified distress following negative expectation, information framing, or clinical interaction. Pain-related outcomes were consistently described as particularly sensitive to communicative influences shaped by the care context [24,28,85,102].

Experimental and clinical studies highlighted the role of symptom vigilance in shaping nocebo responses. Patients encouraged, explicitly or implicitly, to attend closely to bodily sensations were more likely to report increased pain or adverse symptoms even when treatment exposure was unchanged, with symptom monitoring itself functioning as a reinforcing process over time. Within chronic conditions, repeated clinical encounters and sustained exposure to risk-focused or uncertainty-laden communication were associated with cumulative patterns of symptom amplification, particularly among individuals with higher baseline anxiety or distress [15,20,63,76,85,95,102,105].

#### 3.3.2. Medical Procedures and Intervention-Related Contexts

Nocebo effects were also widely examined within medical procedures and intervention-related contexts, including diagnostic investigations and treatment-adjacent encounters. In these settings, nocebo responses were typically identified through increased procedural pain, anticipatory anxiety, distress, or negative symptom reporting in the absence of objective complications, with procedural contexts heightening sensitivity to expectation and communication [15,44,77,128].

Studies examining procedural pain and discomfort demonstrated that anticipatory information and verbal framing prior to interventions strongly influenced patient experience. Warning-focused explanations, emphasis on discomfort, or repeated attention to potential adverse sensations were associated with heightened pain perception and distress even when procedural variables were unchanged. Within diagnostic and monitoring procedures, uncertainty and heightened sensory focus were further shown to amplify symptom reporting, particularly when combined with repeated questioning or reassurance during or after interventions [15,41,43,44,77,108,122,133].

#### 3.3.3. Pharmacological Treatment and Adverse-Effect Reporting

A substantial proportion of the literature examined nocebo effects in the context of pharmacological treatment, particularly through patterns of adverse-effect reporting, treatment discontinuation, and symptom attribution. In these studies, nocebo effects were operationalised as side effects that could not be explained by pharmacological action alone and were closely linked to expectation, information framing, and prior beliefs about medication [5,45,101].

Across experimental, clinical, and review-based studies, adverse-effect reporting was shown to be highly sensitive to how medication risks were communicated. Warning-focused or detailed side-effect disclosures were consistently associated with increased symptom reporting, including in placebo or sham conditions, and were linked to non-adherence and treatment discontinuation in routine care. Adverse expectations were often described as persisting despite reassurance or objective evidence of safety, highlighting the cumulative influence of communication over time [5,7,22,45,101,138].

#### 3.3.4. Procedure-Adjacent and Embodied Care Contexts

Beyond discrete procedures or pharmacological treatment, a subset of the literature examined nocebo effects within procedure-adjacent and embodied care contexts, where physical positioning, sensory focus, and reduced agency appeared to shape vulnerability to distress. In these settings, nocebo effects were described as emerging through the embodied experience of care itself, including environmental cues, physical constraint, and heightened awareness of bodily sensations [41,44].

Within dentistry and other close-contact clinical environments, anticipatory threat, sensory salience, and constrained movement were linked to increased sensitivity to clinician language and behaviour. Routine communication or reassurance could take on heightened significance under conditions of restricted movement or altered control, contributing to increased distress even in the absence of procedural complications. Similar dynamics were described in paediatric and family-centred care, where preparatory communication and reassurance were not uniformly protective and, in some cases, appeared to heighten vigilance when delivered in risk-focused or monitoring-oriented ways [8,41,44,59,63,128].

### 3.4. Clinical and Procedural Contexts Examined

Across the included full-text dataset, nocebo-related mechanisms were examined within a wide range of clinical and procedure-adjacent contexts, spanning direct treatment encounters, consent processes, and healthcare communication environments. A substantial proportion of studies were situated in settings characterised by interpretive uncertainty, particularly pain, somatic symptom reporting, and anxiety-linked bodily sensations, where expectation effects, misattribution, and communication-mediated amplification were commonly examined [2,4,5,38,70,103].

Several studies explicitly located nocebo effects within the ethics and practice of clinical communication, particularly in relation to tensions between respecting autonomy (e.g., risk disclosure) and avoiding iatrogenic symptom amplification. This work positioned nocebo responses within the clinician–patient episode, emphasising interaction, trust, and meaning-making processes rather than purely individual cognitive mechanisms [19,21,93,117,121,139].

A distinct subset of the literature addressed dental and oral-health-adjacent contexts, including explicit consideration of how nocebo mechanisms translate to dentistry and dental decision-making. These studies examined dental-specific framing of expectations, symptom interpretation, and patient agency, alongside broader procedural work highlighting how perceived control and decision capacity may heighten sensitivity to clinician cues [42,44,74,116].

Other studies examined nocebo mechanisms in contexts where information was delivered through written patient materials or institutional messaging, such as anaesthetic information leaflets or precautionary environmental health communications. In these settings, routine phrasing and “protective” messaging were shown to plausibly shape anticipatory symptoms, threat appraisal, and symptom reporting [98,104]. Work in rehabilitation and allied health settings further highlighted how clinicians’ understanding of placebo and nocebo processes influenced how symptoms, recovery, and risk were framed in practice, alongside applied efforts to translate nocebo insights into strategies for reducing avoidable harm in clinical care [23,38,58,62,70,90,97,99,103].

### 3.5. Paediatric Considerations and Developmental Framing

Across the included full-text dataset, explicit paediatric-focused examination of nocebo mechanisms was comparatively limited. However, several studies and applied papers suggested that nocebo-relevant processes in children and young people may operate through distinct developmental, relational, and contextual pathways. Children’s symptom interpretation and distress responses were described as being shaped not only by informational content, but by how information is framed, who delivers it, and how it aligns with children’s evolving capacity to predict, contextualise, and appraise bodily sensations [39,40,129].

A recurring theme concerned developmental understanding and suggestibility. Earlier work demonstrated that children’s reports of pain and distress are highly sensitive to social and verbal cues, with adult reassurance and framing influencing perceived intensity and narrative interpretation of symptoms [39]. Later paediatric-adjacent literature similarly suggested that negative expectations may become embedded rapidly when children are primed to anticipate harm or discomfort, particularly in contexts where sensations are ambiguous or difficult to explain in age-appropriate terms [29,40].

The importance of parental involvement and family-centred framing was also evident. Observational studies in peri-anaesthetic and procedural settings illustrated how clinician communication with both children and parents forms part of the context in which distress and symptom appraisal occur [41]. Work examining adolescents’ and parents’ information needs further highlighted that engagement with health information is variable, shaped by individual preferences, perceived risks, and parental expectations, with implications for how reassurance, warnings, and preparatory explanations are received [41,115].

Within dentistry-adjacent contexts, paediatric-relevant studies examined strategies to reduce anxiety and pain during care, including non-pharmacological interventions and communication approaches. Reviews of paediatric dental care emphasised that pain and anxiety prevention strategies are central to cooperation and treatment experience, providing a setting in which anticipatory threat, symptom vigilance, and communication framing may plausibly influence distress and behaviour [113,128]. Broader paediatric dental commentary further raised concerns about how discomfort and coping are framed in routine practice, highlighting the potential role of expectation management in shaping symptom reporting and distress behaviours [131].

### 3.6. Gaps, Tensions, and Underexplored Areas in the Literature

Across the included literature, several systematic gaps and tensions were evident in how nocebo-related mechanisms have been conceptualised, operationalised, and examined across clinical contexts. Although nocebo effects were widely acknowledged as clinically relevant, the depth and consistency with which contextual, relational, and behavioural factors were empirically examined varied substantially between study types and settings [29,38]. Ongoing uncertainty regarding how nocebo susceptibility varies across individuals and contexts further reflected the fragmented nature of the evidence base [12,49,67,68,100,101].

A prominent gap concerned the over-reliance on experimental pain paradigms and pharmacological trial contexts, relative to the limited examination of complex, real-world care environments. While experimental studies provided important mechanistic insights into expectancy, learning, and symptom amplification, comparatively few studies examined how nocebo-related processes unfold within routine clinical encounters characterised by ambiguity, time pressure, and layered interpersonal dynamics [62,70,84,90,107,132].

A related tension was evident between conceptual recognition of communication as central to nocebo effects and the limited empirical examination of communication practices in situ. Although reviews and commentaries emphasised clinician language, framing, and interaction style, relatively few studies directly observed or analysed communication behaviours within real clinical encounters. Communication was often treated as an inferred modifier rather than as a dynamic, interactional process, with limited fine-grained analysis of how reassurance, choice, and perceived control are enacted during care [19,56,69,93,99,103].

Ethical and practical tensions surrounding informed consent and risk disclosure were also apparent. Several papers highlighted the potential for standard consent practices to inadvertently generate nocebo effects when risk information is delivered without sufficient contextualisation. However, empirical examination of how consent processes are experienced by patients, or how they interact with behaviour support strategies in practice, remained limited [21,59,117,118,121,139].

Another underexplored area concerned contexts of reduced agency or constrained participation, including close-contact procedures, immobilisation, or altered levels of consciousness. While individual studies alluded to heightened vulnerability in such settings, systematic examination of how nocebo mechanisms operate when patients have limited ability to communicate, disengage, or exert control was largely absent, particularly in anaesthesia-adjacent, procedural, and dental contexts [14,42,69,86,98,116].

Finally, the literature demonstrated conceptual fragmentation and inconsistent terminology, with nocebo effects variably described in terms of expectancy, learning, misattribution, communication, or ethical framing. Although this diversity reflects the interdisciplinary nature of the field, several reviews noted the absence of integrative frameworks capable of accommodating both mechanistic and relational accounts, complicating synthesis and translation into clinical guidance [4,12,23,38,48,49,67,68,97,100,101].

## 4. Discussion

To our knowledge, this is the first scoping review to avoid treating nocebo effects as a narrow pharmacological artefact, instead integrating evidence from a heterogeneous body of work through a conceptual framework that foregrounds relational, communicative, and contextual processes.

Across experimental, clinical, and applied studies, nocebo effects have long been recognised as extending beyond drug-related mechanisms, emerging through expectations, prior experience, and the clinical encounter itself [3,7,9,24,45,140]. These expectancy effects are further reinforced through learning and conditioning processes, including observational learning and prior treatment experiences, which influence how bodily sensations are anticipated and interpreted [12,13,78]. Importantly, these mechanisms do not operate in isolation: applied and behavioural studies increasingly highlight the role of clinician communication, reassurance, and informational framing in amplifying or attenuating nocebo responses, situating symptom experience within the dynamics of the clinical encounter and care setting [18,19,101]. Together, this literature supports a conceptualisation of nocebo effects as dynamic, interactional phenomena, shaped not only by what treatments are delivered, but by how information is conveyed and relationships are negotiated within specific clinical environments [2,85].

Rather than operating as discrete or sequential mechanisms, the processes underpinning nocebo effects consistently co-occur and interact across the literature, challenging attempts to categorise them in isolation. Studies examining expectancy frequently implicate learning histories and prior experiences, while those focused on communication often simultaneously engage with ethical considerations around disclosure and consent [93,111,114]. This conceptual entanglement is evident across experimental and applied work, where verbal suggestion, observational learning, and contextual framing are shown to jointly shape symptom anticipation and interpretation [5,10,53,72,94]. Importantly, several reviews and empirical studies explicitly caution against mechanistic accounts that privilege a single explanatory pathway, instead emphasising the relational and situational nature of nocebo responses as they unfold in real clinical encounters [19,98]. Viewed in this way, overlap across conceptual domains is not a methodological weakness but a reflection of how nocebo effects are generated in practice, through the dynamic interplay of expectations, communication, learning, and context rather than through any single isolated mechanism [24].

Across the nocebo literature, expectancy and learning processes consistently emerge as foundational mechanisms through which symptom experience is shaped, providing the substrate upon which communicative and contextual factors later operate [60,79,80]. These expectancy effects are not static but are dynamically reinforced through learning mechanisms, including classical conditioning, prior treatment outcomes, and observational learning, which together shape how bodily sensations are anticipated and interpreted over time [13,16,48,86]. Viewed in this way, expectancy and learning do not merely precede nocebo responses but actively structure how later communication, reassurance, and contextual cues are received, interpreted, and integrated into the patient’s ongoing experience of care [27,60].

Clinician communication does not merely transmit neutral information but functions as an active mechanism through which symptom perception, vigilance, and distress are shaped [2,17,18,55,65]. Communication can therefore act as a contextual cue that amplifies or attenuates nocebo responses, even in the absence of pharmacological change [7,22,64,126]. Importantly, these effects are not confined to explicit warnings or risk disclosure. Subtle linguistic choices, clinician affect, and interactional dynamics have been shown to alter anxiety, expectancy, and symptom monitoring, with downstream effects on pain and distress [6,19,20]. Studies of patient-practitioner interaction consistently demonstrate that communication styles intended to support transparency or reassurance may nevertheless heighten vigilance when delivered without sensitivity to context, prior experience, or perceived control [21,93,120]. Taken together, this literature positions communication not as a passive conduit for information, but as a causative component of the nocebo process itself, operating through relational and contextual pathways rather than through suggestion alone [5,23].

Reassurance is typically framed as an unambiguously benign component of behaviour support, intended to reduce anxiety and promote patient comfort. However, across the nocebo literature, repeated checking-in and verbal reassurance emerge as relational cues that can heighten vigilance rather than diminish it [6,10,58,83]. When attention is repeatedly drawn to bodily sensations, potential discomfort, or the possibility of adverse effects, reassurance may paradoxically amplify symptom monitoring and distress, particularly in individuals already sensitised by prior experiences or expectations [1,25,50]. Experimental and applied studies suggest that such reassurance can function as a signal that threat is present or anticipated, thereby reinforcing negative expectancies even when explicitly intended to provide comfort [46,78,79,80,119,130]. These effects appear especially pronounced when reassurance is delivered repeatedly or reflexively, without sensitivity to context, agency, or the patient’s interpretive frame. Rather than restoring a sense of control, repeated verbal checking-in (e.g., “Are you OK?”) may externalise authority to the clinician and intensify attentional focus on internal states, creating a feedback loop in which distress prompts reassurance, which in turn sustains vigilance and symptom awareness [11,56,57,79,93,127]. Within this “reassurance loop”, communication intended to support coping may inadvertently reinforce helplessness, uncertainty, or threat appraisal, positioning reassurance itself as an active contributor to nocebo responses rather than a neutral or protective intervention [23,75,86,123,124].

The same communicative practices that aim to promote transparency, informed consent, and patient autonomy may inadvertently contribute to symptom misattribution and nocebo-related harm. A growing body of literature highlights how risk information, adverse event labelling, and side-effect framing can shape patients’ expectations and subsequent interpretations of bodily sensations, increasing the likelihood that benign or unrelated symptoms are attributed to treatment [47,51,73,92,125].

Ethical analyses of nocebo effects therefore increasingly question whether conventional models of disclosure sufficiently account for the relational and interpretive dimensions of risk communication. Several authors argue that a narrow focus on informational completeness may overlook how consent encounters are experienced by patients, particularly when uncertainty, power imbalance, or time pressure are present [26,54,66,82,96,106]. Importantly, this reframing does not advocate for paternalism or concealment, but for a more reflexive approach to communication that considers how information is conveyed, when it is delivered, and what interpretive work it invites patients to do. Empirical and conceptual contributions alike suggest that ethically responsible practice requires clinicians to balance transparency with an awareness of nocebo risk, attending not only to the content of disclosure but to its emotional impact and encounter-level effects [52,89]. In this sense, nocebo effects sit at the intersection of communication and ethics, revealing how well-intentioned practices can produce unintended harm when attribution processes are left unexamined.

Nocebo effects may be particularly salient in paediatric contexts, where developmental stage, power imbalance, and reduced agency shape how communication is perceived and internalised. Children rely heavily on external cues to make sense of bodily sensations and clinical encounters, rendering them especially sensitive to clinician language, tone, and non-verbal behaviour, as well as to parental responses and modelling. Empirical studies demonstrate that children’s symptom reports, pain experiences, and emotional responses are strongly influenced by expectation, suggestion, and social learning, often to a greater extent than in adults [39,40,128]. In such contexts, reassurance, warnings, and repeated checking-in may carry amplified meaning, particularly when delivered in environments characterised by uncertainty or perceived threat. Importantly, children’s limited control over treatment decisions and reduced capacity to contextualise risk information may intensify attentional focus on bodily sensations, increasing vulnerability to symptom misattribution and distress [41,129]. These dynamics position paediatric care as a context in which nocebo mechanisms are not only present but potentially magnified, underscoring the need to consider how developmental factors, relational cues, and agency interact to shape children’s experiences of care.

Paediatric inhalation sedation provides a particularly instructive case through which to examine the interaction of nocebo mechanisms, behaviour support, and communication in dental care. In this context, reassurance and supportive language are delivered to children whose consciousness, sensory processing, and agency are altered by sedation, potentially changing how verbal cues are interpreted and internalised [116,129]. Contemporary behaviour support frameworks in paediatric dentistry explicitly recognise communication, reassurance, and interactional style as active interventions rather than neutral adjuncts, while also acknowledging that their effects are highly context dependent [141].

The physical constraints associated with dental sedation, such as nasal masks, restricted movement, and close clinical monitoring, may further heighten attentional focus on bodily sensations and perceived threat, particularly in children with pre-existing dental anxiety [25,40,74]. Under these conditions, repeated checking-in and reassurance, though well intentioned and widely recommended within paediatric dental behaviour guidance frameworks, may paradoxically reinforce vigilance and distress rather than comfort when delivered without sensitivity to context or perceived control [41,93,98]. This risk may be amplified in children undergoing prolonged or repeated courses of care, where cumulative experiences of dependency and uncertainty can erode coping capacity over time [142]. Viewing paediatric sedation through a nocebo-informed lens therefore highlights how behaviour support strategies, central to both non-pharmacological and pharmacological management, may function differently under altered states of consciousness, underscoring the need to consider not only what is said to children, but how, when, and in what relational context it is conveyed [17,143].

Dentistry provides a particularly informative applied lens through which to examine nocebo effects, given its reliance on anticipatory communication, repeated procedures, and routine management of pain and anxiety across the life course. Dental encounters often involve iterative exposure to treatment environments in which expectations, prior experience, and clinician language are continually reinforced, allowing nocebo processes to accumulate rather than remain episodic [42,59] and commentaries within dental literature increasingly recognise that adverse symptoms and treatment intolerance cannot be fully explained by procedural factors alone, but are shaped by communication, attribution, and patient interpretation [8,63,131]. Empirical work in dental pain and anxiety further demonstrates that expectations and contextual cues substantially modulate symptom experience, including in routine and low-risk procedures [110,133]. Recent syntheses confirm that nocebo effects are both relevant and observable within dentistry, underscoring the discipline’s value as a real-world setting in which relational and contextual mechanisms of nocebo can be examined and addressed [44,74].

This review has several strengths. It draws on a broad and multidisciplinary body of literature, integrating experimental, clinical, ethical, and applied perspectives to develop a conceptually coherent account of nocebo effects as interactional phenomena shaped by the care setting. By adopting an interpretive scoping approach, the review moves beyond enumeration to identify patterns, overlaps, and mechanisms that are often examined in isolation, offering a framework that is clinically meaningful across settings. However, several limitations should be acknowledged. As with all scoping reviews, the synthesis is necessarily interpretive rather than causal, and the heterogeneity of included study designs precludes formal assessment of effect size or comparative strength of mechanisms. The review is also limited by reliance on published literature, which may underrepresent negative or null findings, and by variable depth of reporting across included studies. Nonetheless, these limitations reflect the current state of the field and underscore the need for future empirical work that explicitly examines how nocebo mechanisms interact within real-world clinical encounters.

Recognising nocebo effects as interactional phenomena embedded in routine care carries important implications for clinical practice, training, and future research. Rather than treating communication as an adjunct to technical care, these findings suggest a need for greater reflexivity around how language, reassurance, and behaviour support are enacted across clinical encounters, particularly in settings characterised by vulnerability or reduced agency. Training initiatives that foreground communication as an active clinical skill, rather than an intuitive or benign background process, may help clinicians become more aware of how expectations and vigilance are shaped in practice. From a research perspective, future work would benefit from moving beyond isolated mechanism studies toward designs that examine how expectancy, learning, communication, and context interact over time, including across repeated encounters. Qualitative and mixed-methods approaches may be particularly valuable in capturing how patients interpret reassurance and support in situ. Taken together, this review highlights the importance of shifting from a purely informational model of care toward one that attends explicitly to the relational dynamics through which nocebo effects are generated and sustained.

## 5. Conclusions

This scoping review demonstrates that nocebo effects are conceptualised across the literature not as isolated pharmacological artefacts, but as relational and communicative phenomena emerging through expectation, learning, and clinical interaction. Verbal and non-verbal communication, including reassurance, framing, and interactional style, are consistently implicated in shaping negative expectations and symptom perception.

The review highlights that reassurance and behaviour support, while central to clinical care, remain variably theorised and insufficiently examined in relation to nocebo processes. This is particularly relevant in paediatric and procedural contexts, where reduced agency and heightened vulnerability may influence how communication is received and internalised. Clinically, these findings emphasise that communication should not be viewed as neutral, but as an active component of care capable of shaping patient experience. Greater attention to how reassurance, expectation-setting, and repeated checking-in are delivered may help minimise unintended distress, while maintaining transparency and trust within the clinical encounter.

## Figures and Tables

**Table 1:** Example Ovid MEDLINE search strategy used to identify studies for the review.

exp Nocebo Effect/or nocebo.mp. or negative suggestion*.mp. or “expectation induced symptom*”.mp. or anticipatory anxiety.mp. or threat cue*.mp. or “communication induced anxiety”.mp. or suggestibility.mp. or acquiescence bias.mp. or alarming medical jargon.mp. or (Overemphasi* adj2 side effect*).mp. or “reassurance induced distress”.mp. or “reassurance induced anxiety”.mp. or “pain talk”.mp. or warning phrase*.mp. or “triviali*ing concer*”.mp. [mp=title, book title, abstract, original title, name of substance word, subject heading word, floating sub-heading word, keyword heading word, organism supplementary concept word, protocol supplementary concept word, rare disease supplementary concept word, unique identifier, synonyms, population supplementary concept word, anatomy supplementary concept word] exp Child/or exp Pediatrics/or pediatric*.mp. or paediatric*.mp. or child*.mp. [mp=title, book title, abstract, original title, name of substance word, subject heading word, floating sub-heading word, keyword heading word, organism supplementary concept word, protocol supplementary concept word, rare disease supplementary concept word, unique identifier, synonyms, population supplementary concept word, anatomy supplementary concept word] exp Pain/or (exp Fear/and exp Needles/) or pain.mp. or distress.mp. or reassurance paradox.mp. or “procedure related anxiety”.mp. or “treatment related anxiety”.mp. or “medical procedural anxiety”.mp. or needle fear.mp. or “fear of needle*”.mp. or “fear of injection*”.mp. or “coping language”.mp. or “parent child communication”.mp. or “clinician child communication”.mp. [mp=title, book title, abstract, original title, name of substance word, subject heading word, floating sub-heading word, keyword heading word, organism supplementary concept word, protocol supplementary concept word, rare disease supplementary concept word, unique identifier, synonyms, population supplementary concept word, anatomy supplementary concept word] 2 and 3 1 and 4 1 and 3 5 or 6 exp Injections/or exp Anesthesia/or exp Emergency Medical Services/or exp Nurse-Patient Relations/or exp Magnetic Resonance Imaging/or exp Pain Management/or exp Suggestion/or exp Physical Therapy Modalities/or exp Orthopedic Procedures/or (exp Dental Care/or exp Dental Anxiety/) (injection* or venepuncture or anesthesia or anaesthesia or preoperative communication* or emergency care or emergency treatment or nursing communication or “nurse patient communication” or magnetic resonance imaging or mri or imaging procedure* or pain management clinic* or suggestion therap* or physiotherap* or physical therap* or musculoskeletal care or dental care or dental treatment).mp. [mp=title, book title, abstract, original title, name of substance word, subject heading word, floating sub-heading word, keyword heading word, organism supplementary concept word, protocol supplementary concept word, rare disease supplementary concept word, unique identifier, synonyms, population supplementary concept word, anatomy supplementary concept word] 8 or 9 7 and 10 *nocebo effect/and nocebo.ti. 11 or 12 limit 13 to English language

**Table 2:** Data extraction fields used to record included studies.

Author(s) Year of publication Title of publication Aim(s) or purpose of the study Source of publication (e.g., journal, report, thesis) Country of corresponding author(s) Study design (e.g., experimental, qualitative, review, theoretical) Study population and setting (including age group, where applicable) Clinical or experimental context (e.g., medical, dental, procedural, laboratory-based) Conceptualisation of nocebo effects or negative expectancy Type of communication or behavioural interaction examined Outcome(s) or response(s) assessed (e.g., anxiety, symptom perception, distress) Key findings relevant to the review questions Implications for behaviour support or clinical communication

**Table 3:** Conceptual Groupings of included Nocebo Literature.

Conceptual Grouping	Description of Focus	Study Designs Represented	Clinical Contexts	Number of Studies	References
Expectancy-driven symptom amplification	Nocebo effects framed as expectancy-mediated changes in symptom perception	Experimental, Randomised controlled trials, reviews	Pain, pharmacology, procedures	50	[1,3,4,5,7,8,9,10,11,16,18,24,25,26,27,28,29,38,46,47,48,49,50,51,52,53,54,55,56,57,58,59,60,61,62,63,64,65,66,67,68,69,70,71,72,73,74,75,76,77]
Learning, conditioning and prior experience	Nocebo responses acquired via conditioning, memory, and social learning	Experimental, mechanistic	Pain, procedural settings	29	[1,5,11,12,13,14,15,24,48,57,61,66,69,71,75,78,79,80,81,82,83,84,85,86,87,88,89,90,91]
Communication-mediated and relational processes	Nocebo effects emerging through clinician communication and interaction	Observational, qualitative, applied	Clinical consultations, dentistry, anaesthesia	36	[1,3,5,10,16,17,18,19,20,21,26,29,38,41,53,59,63,65,68,70,71,72,74,85,90,92,93,94,95,96,97,98,99,100,101,102,103]
Adverse-event attribution and risk communication	Misattribution of symptoms to treatment following risk disclosure	Reviews, trials, ethics papers	Consent, pharmacology, procedures	21	[4,5,6,29,38,43,45,46,49,56,62,69,100,101,104,105,106,107,108,109,110]
Ethical, informational and consent-related framing	Tensions between autonomy, disclosure, and harm	Ethics, commentary, reviews	Informed consent contexts	33	[10,17,21,22,23,46,52,59,62,73,74,96,97,99,101,103,105,111,112,113,114,115,116,117,118,119,120,121,122,123,124,125]
Paediatric and developmentally specific contexts	Nocebo effects in children and adolescents	Observational, reviews	Paediatrics, dentistry, concussion	13	[15,39,40,41,82,115,126,127,128,129,130,131,132]
Dentistry-specific nocebo literature	Nocebo phenomena in dental pain, anxiety, and communication	Reviews, trials	Dentistry	8	[8,42,44,59,63,74,131,133]

## Data Availability

The data presented in this study are available on request from the corresponding author. As this study is based on the synthesis of previously published literature, no new datasets were generated or analysed.
